# The quantum physics of intraductal papillary mucinous neoplasm of the pancreas

**DOI:** 10.1093/bjsopen/zrac082

**Published:** 2022-06-17

**Authors:** Giovanni Marchegiani, Giampaolo Perri, Roberto Salvia

**Affiliations:** Department of General and Pancreatic Surgery, Verona University Hospital, Verona, Italy; Department of General and Pancreatic Surgery, Verona University Hospital, Verona, Italy; Department of General and Pancreatic Surgery, Verona University Hospital, Verona, Italy

In 1997, a 54 year old woman underwent pancreatoduodenectomy at Verona University Hospital for a 3-cm periampullary cyst with a solid component. The final pathology reported a peripheral intraductal papillary mucinous neoplasm (IPMN) with low-grade dysplasia. The pancreatic margins were free from neoplastic cells.

After 22 years of clinical and radiological surveillance, the same person, now 76 years old, presented at the same Hospital with increased CA19-9 levels and an abdominal MRI showing a 4-cm cyst, with an enhancing solid component, in the retro-portal lamina area next to the superior mesenteric vessels. A ‘recurrence’ of the original IPMN was found in the context of a small fragment of uncinate process, left in place during the first surgery. Despite the concerning radiographical and serological features, final pathology found an IPMN of intestinal type with—again—only low-grade dysplasia.

The scientific literature on IPMNs has come a long way since 1997. Recently, the largest series of IPMNs ever resected by a single referral centre was published^1^. In this study, among 1439 patients, the timing of resection was categorized according to final pathology: too early (low-grade dysplasia); too late (invasive cancer); and timely (intermediate- and high-grade dysplasia). Only 34 per cent of the ‘timely’ group had radiographical criteria of suspicion, and 25 per cent of the ‘too late’ group was in a watch-and-wait policy before resection. The authors therefore conclude that ‘the “blind” pursuit of a watch-and-wait policy cannot be justified’. While the authors must be commended for their invaluable contribution to the field, messages like this are quite misleading. The entire frame of this syllogism is in fact based on a sequential, time-dependent vision of the IPMNs progression, and assuming the ‘blindness’ of surveillance policies.

Some recent findings suggested different tumorigenic pathways and independent polyclonal origins for IPMNs^2,3^. Despite the knowledge regarding disease natural history remaining limited, the old straightforward adenoma-to-carcinoma progression concept has been already challenged^3^. From a tumorigenic standpoint, IPMNs are not colonic polyps, as well as pancreatectomies are also not endoscopic polypectomies. The overall major postoperative morbidity and mortality of patients undergoing pancreatic resection remains relatively high. To suggest liberal policies for surgical resection seems anachronistic, especially in times where the scientific community struggles to identify targets of safe follow-up reduction over time or even its complete discontinuation in cases of ‘trivial’ IPMNs to relieve the healthcare from the socioeconomic burden of such high-prevalent disease^4^. While it may be true that the fate of all IPMNs is to eventually become invasive, as malignant risk seems to increase with time, it is also true that time is relative (at least to the patient’s age), and that zero risk will never be achieved as long as the general population has a pancreas. The case hereby presented is a clear example of time ‘relativity’, as the IPMN histology remained identical after 22 years, while everything changed around it (the patient, the surgical technique of pancreatoduodenectomy, the surgeon’s hair colour).

Speaking of relativity, quantum physics could be applied also to explain another important conundrum of surgery and IPMNs; how to identify the ‘timely’ target of surgical resection? This also has crucial ethical and legal concerns. Some might speculate that to resect a pre-malignant neoplasm is timely by definition. To surgically avoid the development of overt cancer cannot be legally prosecuted as it is not ‘too early’ *per se*. In the above-mentioned study^1^, invasive cancer was ruled out in 88 per cent of patients using current radiographical features of suspicion suggested by guidelines. Separating low- from high-grade dysplasia using clinical features seems more utopistic, as only one-third of the ‘timely’ group had radiographical criteria of suspicion. From this standpoint, IPMNs unfortunately resemble the Schrödinger’s cat^5^ (*Fig. 1*). In this famous paradox, a cat is penned up in a steel chamber, along with a Geiger counter with a tiny bit of radioactive substance, so small, that perhaps over the hour one of the atoms decays, but also, with equal probability, perhaps none. If it happens, the counter tube discharges and through a relay releases a hammer that shatters a small flask of poison. The indeterminacy originally restricted to the atomic domain becomes transformed into macroscopic indeterminacy, which can then be resolved only by direct observation. Unless the observer (surgeon) opens the box (patient’s belly), the cat (IPMNs) is both alive (low-grade) and dead (high-grade) at the same time.

**Fig. 1:**
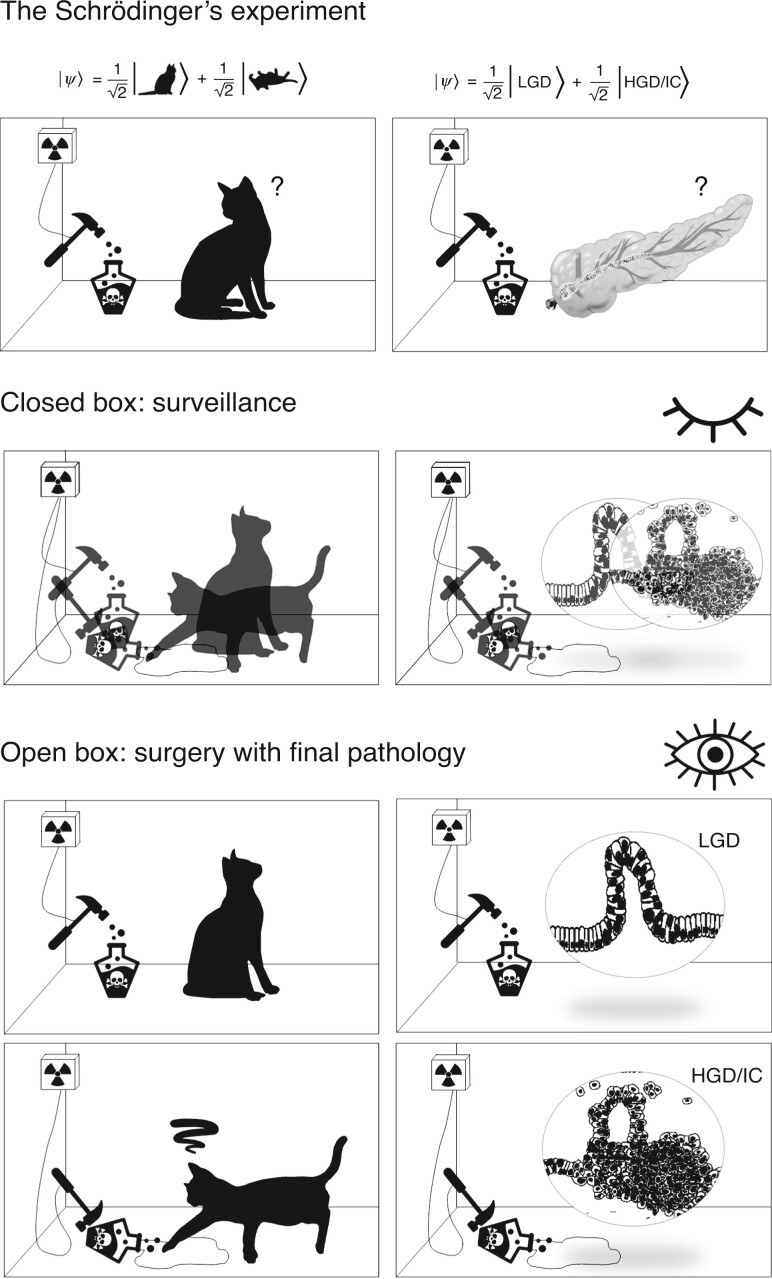
**The Schrödinger’s cat (left) and the Schrödinger’s pancreas (right)** LGD, low-grade dysplasia; HGD, high-grade dysplasia; IC, invasive cancer.

Surgical series alone are not able to crack the enigma as they provide no information on IPMN biology. At present, observational studies focusing on cases ‘crossing over’ from observation to surgery may identify further dynamic predictors of malignant transformation^6^ with the aim of treating high-grade dysplasia before the occurrence of cancer.

Hopefully, the development and validation of reliable biomarkers will represent the next game changer in clinical practice. Several studies already found promising associations between histopathological diagnosis and genetic, epigenetic, gene expression, and protein biomarkers in serum or cyst fluid^7,8^; however, as has happened historically for clinical features, the associations between the marker of interest and the histological endpoint were calculated only in resected patients with a known pathological diagnosis by most of these studies. Little is known about biomarker performance in patients kept under surveillance for a presumed IPMN^9^. It is still unclear what duration of follow-up without cancer development is necessary to state that low-risk disease was present at the time of biomarker assessment. In current clinical practice, biomarkers still do not allow for the selection of the correct candidate for surgery, observation, or neither^10^.

## Disclosure

The authors declare no conflict of interest.
